# Design and Implementation of a Flexible Electromagnetic Actuator for Tunable Terahertz Metamaterials

**DOI:** 10.3390/mi15020219

**Published:** 2024-01-31

**Authors:** Shengru Zhou, Chao Liang, Ziqi Mei, Rongbo Xie, Zhenci Sun, Ji Li, Wenqiang Zhang, Yong Ruan, Xiaoguang Zhao

**Affiliations:** 1 School of Instrumental Science and Opto-Electronics Engineering, Beijing Information Science Technology University, Beijing 100192, China; 2 Department of Precision Instrument, Tsinghua University, Beijing 100084, China; 3 Key Laboratory of MEMS of the Ministry of Education, Southeast University, Nanjing 210096, China; 4 College of Engineering, China Agricultural University, Beijing 100083, China; 5 State Key Laboratory of Precision Measurement Technology and Instruments, Tsinghua University, Beijing 100084, China; 6 Beijing Advanced Innovation Center for Integrated Circuits, Tsinghua University, Beijing 100084, China

**Keywords:** electromagnetic actuator, MEMS, flexible polyimide, tunable terahertz metamaterial

## Abstract

Actuators play a crucial role in microelectromechanical systems (MEMS) and hold substantial potential for applications in various domains, including reconfigurable metamaterials. This research aims to design, fabricate, and characterize structures for the actuation of the EMA. The electromagnetic actuator overcomes the lack of high drive voltage required by other actuators. The proposed actuator configuration comprises supporting cantilever beams with fixed ends, an integrated coil positioned above the cantilever’s movable plate, and a permanent magnet located beneath the cantilever’s movable plate to generate a static magnetic field. Utilizing flexible polyimide, the fabrication process of the EMA is simplified, overcoming limitations associated with silicon-based micromachining techniques. Furthermore, this approach potentially enables large-scale production of EMA, with displacement reaching up to 250 μm under a 100 mA current, thereby expanding their scope of applications in manufacturing. To demonstrate the function of the EMA, we integrated it with a metamaterial structure to form a compact, tunable terahertz absorber, demonstrating a potential for reconfigurable electromagnetic space.

## 1. Introduction

Micro actuators are crucial components in microelectromechanical systems (MEMS) and have been used in a wide range of applications, from micromirrors to micropumps [1]. Actuators can be categorized based on their driving mechanisms, including electrostatic [2], piezoelectric [3], thermal [4], and electromagnetic [5], among others. The operation for electrostatic actuators originates from electrostatic forces between two oppositely charged electrode plates. However, the generation of sufficient electrostatic forces requires high driving voltage and leads to a non-linear response, limiting the magnitude of displacement on the order of tens of micrometers [6,7]. The thermal actuators operate based on the thermal expansion under excitation of Joule heating and may exert large forces and displacement. However, the response speed of thermal actuators is relatively slow, and the energy conversion efficiency is low since there is energy transferring in the electric, thermal, and mechanical domains [8]. Piezoelectric actuators are empowered by the voltage-controlled strain in piezoelectric materials, such as lead zirconium titanate (PZT), aluminum nitride (AlN), etc. For example, the utilization of PZT, a well-established piezoelectric ceramic material, has paved the way for the development of commercial micropumps [9]. Nevertheless, the performances of the piezoelectric actuator (PEA) driven systems were significantly degraded due to dynamic issues, namely hysteresis and creep effects [9,10,11]. The electromagnetic actuator (EMA) offers several advantages, including large displacement and immunity to nonlinear effects [12]. Additionally, EMA exhibits rapid response and exceptional precision.

EMA is one of the optimal choices for achieving a large driving force with compact size [13]. However, the manufacture of electromagnetic actuators is complicated, and requires inductive coils to produce magnetic flux [14,15]. The majority of EMAs commonly utilize silicon as the substrate material. For example, Jiang B et al. used a silicon-based manufacturing process to fabricate a two-dimensional micromirror that is driven at a voltage of 5 V [16]. However, the fabrication process of silicon-based actuators is costly and complicated. In addition to this, rigid EMA tends to be bulky and can cause damage to the human body when it is operated in close proximity; traditional EMA promotes the development of flexible EMA [17]. Alternatively, polymers possessing outstanding mechanical, thermal, and chemical properties provide a choice for the substrate material [18,19]. Chia-Yen Lee et al. electroplated copper coils on a polyimide(PI) film, resulting in a displacement of 150 μm [20]. Z. Wang et al. made a flexible soft robot using PI electroplating and re-transfer substrate, but the magnetic field generated by the magnetic particles of the robot was only 120 mT [21]. Sun et al. designed a tunable metasurface that continuously controls the air gap by introducing a conventional EMA, which is a voice coil motor (VCM). The VCM is bulky and exhibits a slow response time [22]. In this paper, a flexible PI material with excellent mechanical properties and high-temperature resistance is used as an actuator structure, and a massive permanent magnet is used to generate a larger magnetic field. Micromirrors based on MEMS technology have been widely used in optical display, optical communication, and other applications [23]. However, traditional micromirrors are mainly driven by piezoelectric ceramics and VCM. The EMA possesses high integration and fast response speed, making it a potential replacement for traditional VCM. Integrating the metasurface absorber into the microlens system enables applications in optics and imaging.

We developed a multi-physics model to design and optimize the planar square spiral coil on the flexible substrate. The magnetic flux density distribution induced by the planar coil is determined using simulation, and the location of the greatest is pinpointed. Additionally, a comprehensive simulation is performed to optimize the magnetic flux density and the wire, thickness, and number of turns in the planar coil. We fabricated the tunable metamaterials that typically require a specific displacement capability to effectively manipulate electromagnetic waves [24,25]. The actuator has a large displacement, and 250 μm enables metamaterials to regulate electromagnetic waves in different operating states, facilitating the control and fine-tuning of optical, acoustic, or electromagnetic wave behavior.

## 2. Theory and Design

**Figure 1:**
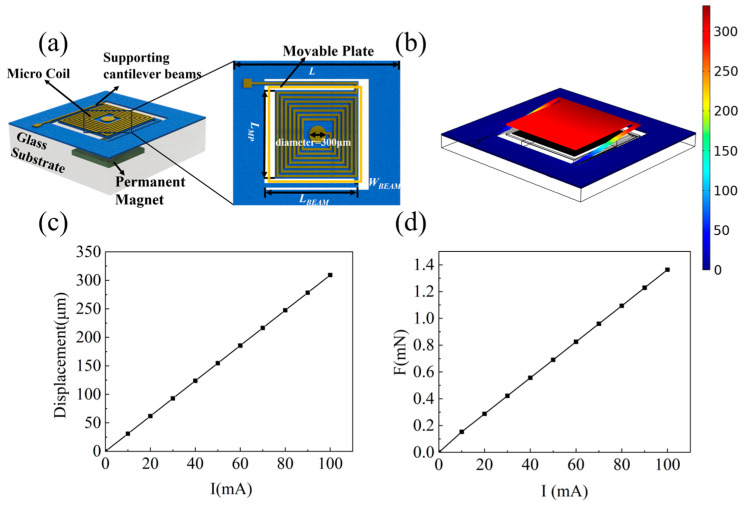
Operation principle of the proposed electromagnetic actuator (EMA). (**a**) Design of the EMA; (**b**) simulated deformation of the EMA structure; (**c**) displacement of the movable plate at different currents; (**d**) electromagnetic force at different currents.

The proposed EMA consists of supporting beams, a movable micro coil patterned on a plate, and a permanent magnet, as shown in Figure 1a. We employed PI, which is a polymer material commonly used in flexible circuits, as the material for the supporting beams and plate. The micro coil is copper wires patterned on both sides of the plate. A permanent magnet is embedded in the glass substrate beneath, generating a uniform static magnetic field for EM actuation. The overall area of the actuator is 8 mm × 8 mm, and the movable plate is 4 mm × 4 mm. The thickness of the PI is 60 μm. The linewidth of the metal wire in the micro coil is 10 μm. The number of turns in the planar spiral is 26. The separation distance between the magnets and the coil is approximately 2.3 mm, which is controlled by the thickness of the glass. Detailed parameters are listed in Table 1.

As shown in Figure 1b, we built the multi-physical model EM actuator and evaluated the deformation of the structure. In the numerical model, the ends of the cantilevers in the actuator are fixed on the glass substrate. An external direct current (DC) voltage source is connected to the actuator. When there is a current applied to the planar micro coil, the induced EM force pushes the plate vertically and bends the cantilevers. The displacement of the plate and the induced EM force increase linearly as the current increases from 0 to 100 mA, as shown in Figure 1c,d. The displacement of the movable plate is approximately 300 μm with current ranging from 0 to 100 mA. The direction of the displacement and force is related to the direction of the current. For instance, if the polarity of the induced magnetic field by the planar micro coil is the same as that of the permanent magnet, the electromagnetic force overcomes the restoring force of the cantilever beam, thereby driving the MEMS actuator upward.

To understand the generation of the EM force, we performed a theoretical analysis of the magnetic field. The field analysis of the planar square spiral coil, following Biot–Savart’s law, involves the generation of magnetic flux when current flows through the coil. The magnetic flux predominantly concentrates within the central region of the coil. We denote the current flowing through the planar spiral coil as *I* and consider an arbitrary section of the current element represented by *Idl*. Assuming a point *R* located at a distance from the current element, with an angle *θ* between the point and the direction of motion of the current element, the magnetic field generated by the current element at arbitrary point P in space can be expressed as [26]:

(1) $$\mathrm{d}B=\frac{\mu_{0}}{4\pi }\frac{I\mathrm{d}l\sin\theta }{R^{2}}$$

where *μ*_0_ (= 4*π* × 10^−7^ H/m) is the permeability in a vacuum, and *B* is induced magnetic flux density; for a wire with a length of *L*, the overall induced magnetic field is as follows:

(2) $$\vec{B}=\int \mathrm{d}\vec{B}=\frac{\mu_{0}}{4\pi }\int_{L}\frac{I\mathrm{d}l\times R}{R^{3}}$$

The electromagnetic force arises from the interaction between an external magnetic field *H*, an electric current *I*, and a magnetization *M*. When the volume of a permanent magnet is *V*, and it has a constant magnetization of *M*, it generates Lorentz force *F* [13]:

(3) $$\mathrm{d}F=\nabla \left(M\cdot H\right)\mathrm{d}V=M\cdot \nabla H\mathrm{d}V$$

When a permanent magnet is vertically magnetized, it generates a vertical electromagnetic force *F_Z_*. The resultant electromagnetic force in the vertical direction is obtained. The resultant electromagnetic force in the vertical direction is obtained [27,28].

(4) $$F_{Z}=\int_{V}M_{Z}\frac{\mathrm{d}B_{Z}}{\mathrm{d}Z}\mathrm{d}V$$

where *B_z_* represents the vertical component of magnetic flux density generated by a planar coil upon energization, and *M_z_* denotes the vertical magnetization component of a permanent magnet after being magnetized.

The electromagnetic force calculation is made using finite element analysis. By applying a current ranging from 0 to 100 mA to the coil, the actuator generates an electromagnetic force. This force generates the displacement of the movable plate, resulting in the deformation of the beam. The relationship between the displacement of the movable plate and the increase in current is depicted in Figure 1d. At a current of 100 mA, the corresponding electromagnetic force is approximately 1.38 mN.

**Figure 2:**
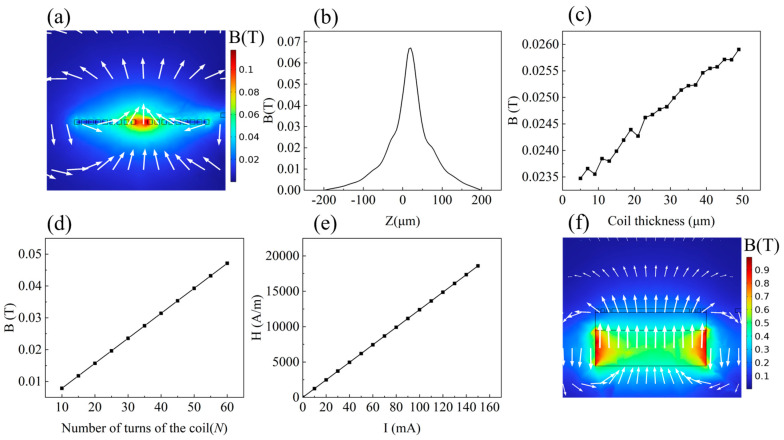
Analysis of the magnetic flux density of the micro coil. (**a**) The magnetic flux density generated by the planar coil; (**b**) the influence of vertical distance of the planar coil on magnetic flux density; (**c**) the influence of coil thickness on magnetic flux density; (**d**) the influence of the number of turns on magnetic flux density; (**e**) relation between magnetic field strength and the number of turns in the micro coil; (**f**) the magnetic flux density generated by a coil superimposed on a permanent magnet.

The electromagnetic force is determined by various factors, including coil parameters, the applied current, and the structure of permanent magnets. When an electric current flows through a coil, it generates a magnetic flux density, according to Ampere’s law. This theorem states that the magnetic flux enclosed by the current is a closed loop. A current of 250 mA is applied to the coil; at the central region of the coil, the magnetic field lines align and combine, resulting in the highest magnitude of magnetic field strength, as shown in Figure 2a. We probed the magnetic flux density along the *z*-axis in the simulation and observed that the induced magnetic field peaks at the plane of the coil and decays dramatically when the position moves away from the plane, as shown in Figure 2b. According to Equation (2), the magnetic flux density decreases following the inverse cubic relation as the distance increases. Consequently, the central region of the coil is the optimal position for leveraging the magnetic force, and the external magnetic field may be placed close to the center of the micro coil [29]. The relationship between coil thickness and magnetic flux density involves many factors, including coil geometry and material properties. The increase in thickness and width of the copper wire in the micro coil leads to an increase in its current-carrying capacity, resulting in a stronger magnetic flux density. A current of 250 mA is applied to the coil. Figure 2c,d provides a simulation that depicts the correlation between wire thickness and turns per coil and the magnetic flux density of the planar coil. 

The magnetic flux density *B* shows a proportional increase with the coil thickness. In the same vein, the number of turns in the plane coil directly impacts the magnetic flux density and the resulting electromagnetic force.

However, the negative impact of increasing the thickness of the flat coil wire on the consistency of the plating process and the overall process cost should also be considered. Similarly, too many turns can lead to increased coil density, which poses challenges during manufacturing and increases costs. According to the magnetic moment of a planar coil, the generated magnetic flux density is directly proportional to the current passing through it. However, excessive current can damage coils primarily due to resistive heating in the coil’s conductive material and associated thermal effects. To prevent such damage, it is recommended to limit the current passing through the coil to a range of 0 mA to 100 mA. Figure 2e illustrates the relationship between magnetic field strength and current intensity. As just discussed, the magnetic field has a linear relationship with the current.

Permanent magnets are a crucial part of generating the EM force [30]. The magnetic field generated by the coil superimposed on the permanent magnet is shown in Figure 2f below, and the permanent magnet material used is NdFeB (*N52*). The magnitude of remanence (*Br*) is 1.42–1.47 T, and a current of 250 mA is applied to the coil. It can be seen that the addition of permanent magnets significantly increases the magnetic field strength, thus making the actuator produce greater displacement.

To investigate the effect of cantilever parameters on the EMAs response, we performed a simulation of the central plate displacement by varying the geometric parameters of the cantilever. The linewidth of the metal wire in the micro coil is 10 μm, and the number of turns in the planar spiral is 26. The current through the coil is 100 mA.

**Figure 3:**
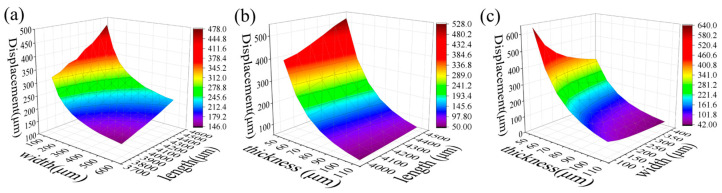
Analysis of the impact of the cantilever beam parameters on the actuator performance. (**a**) Influence of length (*L_BEAM_*) and width (*W_BEAM_*) of cantilever beam on actuator displacement; (**b**) influence of length (*L_BEAM_*) and thickness (*P*) of cantilever beam on actuator displacement; (**c**) influence of thickness and width of cantilever beam on actuator displacement.

Figure 3a demonstrates the effect of simultaneous changes in the length and width of the cantilever beam on the change of actuator displacement. In this calculation, the thickness of the beam is fixed at 60 μm. We gradually increase the length of the cantilever beam from 3700 μm to 4600 μm and vary the cantilever width from 100 μm to 60 μm. The results demonstrate that the displacement of the movable plate increases as the length of the cantilever beam increases, and its width decreases since the stiffness of the beam decreases. However, a longer cantilever beam will result in the increased size of the device, and the length of the beam is set to be 4300 μm under the condition that the overall size is 8 mm × 8 mm. The effect of the PI thickness is also investigated. As shown in Figure 3b, the width of the cantilever beam was fixed at 200 μm while gradually increasing the length from 4000 μm to 4500 μm, and the thickness increased from 60 μm to100 μm. The results demonstrate that a thinner cantilever beam exhibits reduced stiffness, consequently leading to an increased actuator displacement. Consider that the thinner the actuator is made, the closer it is to the underlying permanent magnet, the greater the magnetic field strength; combined with the process and production difficulty, the beam thickness is set to 60 μm. When the length of the fixed cantilever beam is 4300 μm, the width of the cantilever beam gradually increases from 100 μm to 400 μm, and the thickness gradually increases from 60 μm to 100 μm. The results (Figure 3c) indicate that the decreased thickness and width lead to enlarged displacement with increased fabrication challenges and degraded stability. As a compromise, the width of the cantilever beam is chosen to be 200 μm. According to the simulation, the current of 100 mA leads to 325 μm displacement.

## 3. Fabrication

**Figure 4:**
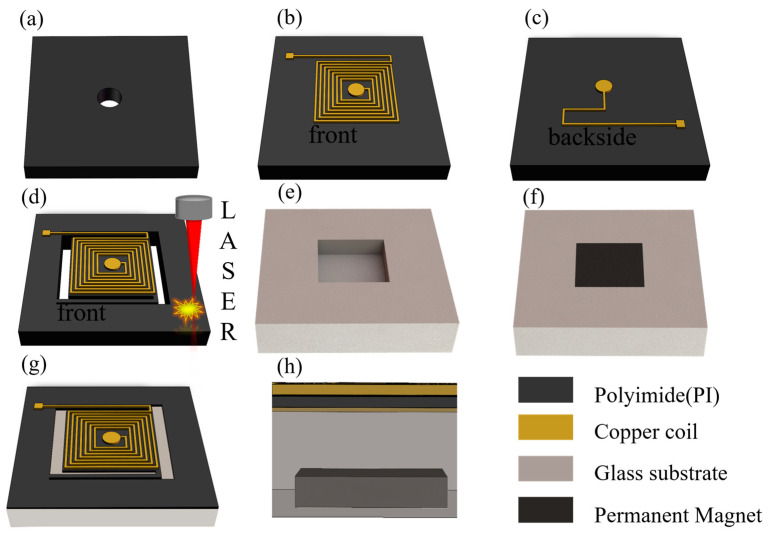
Fabrication process flow of the EMA. (**a**) PI film punching; (**b**) the front layer of coil plating; (**c**) through hole connection, back layer coil plating; (**d**) actuator structure cutting; (**e**) glass substrate cutting; (**f**) permanent magnets are embedded in the glass substrate; (**g**) the actuator is connected to the substrate; (**h**) cross-section view of the EMA.

The fabrication process of the EMA is shown in Figure 4, including the manufacturing of the movable structure and the manufacturing of the substrate. 

The fabrication of the movable structure starts with a PI film with a thickness of 60 μm. In the center of the PI film, a hole with a diameter of 300 μm is perforated for the interconnection of the front and back coils, as shown in Figure 4a. Subsequently, the through holes are filled with copper, and a front copper coil with a thickness of 30 μm is electroplated onto the PI film. After electroplating, wet etching is performed to form the planar square spiral structure, as shown in Figure 4b. The back coil of the PI film is then electroplated and etched to create another 30-μm-thick coil, as shown in Figure 4c. At this point, the coils on both sides of the PI film are patterned. After the coils are patterned, the device is cut by laser ablation to form the cantilevers and movable plate, as shown in Figure 4d. The other part of the device involves the fabrication of a glass substrate with a cavity and permanent magnets. As shown in Figure 4e, this process includes laser ablation of the glass wafer to form a cavity for the encapsulation of the permanent magnet. The cavity has an area of 3 mm × 3 mm and a depth of 0.3 mm. The permanent magnet is then inserted into the cavity and sealed for the assembly, as shown in Figure 4f. Finally, the movable part of the EMA is attached to the glass substrate with a permanent magnet using an adhesive bonding approach, as shown in Figure 4g below. The cross-section of the assembled EMA is shown in Figure 4h.

## 4. Results and Discussion

**Figure 5:**
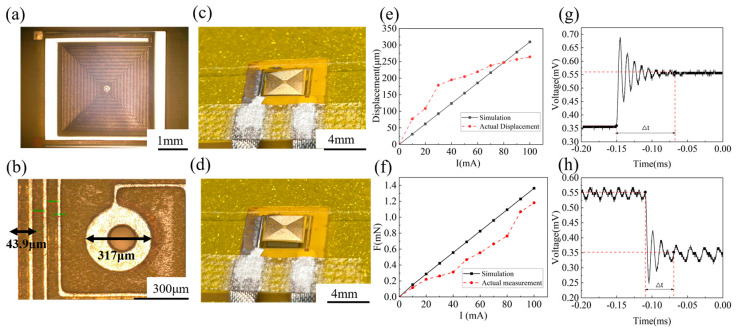
Characterization of the EMA. (**a**) Electron microscope image of the actuator; (**b**) coil center through hole details; (**c**) structure diagram of the actuator without current; (**d**) actuator motion under 70 mA current; (**e**) the displacement depends on the impressed current from 0 to 100 mA; (**f**) the electromagnetic force varies from 0 to 100 mA with an impressed current; (**g**) time response when the actuator is “on”; (**h**) time response when the actuator is “off”.

The optical microscope image of the fabricated EMA is shown in Figure 5a below. The distance between the adjacent wires in the micro coil, as measured by the microscope, is 43.9 μm, while the width of the wire is 10.6 μm. As shown in Figure 5b, the hole connecting the two layers of coils is 317 μm. The resistance of the actuator is measured by a multimeter (UT89XE, UNI-T, Dongguan, China), and the measured resistance is compared with the simulation results obtained by COMSOL Multiphysics 5.6. Analog coil resistivity set to 1.72e^−8^ ohm*m and conductivity set to 5.998e^7^ S/m. The actual measurement coil resistance is 28.3 Ω, and the simulation value is 12.939 Ω. The difference in resistance can be attributed to the presence of contact resistance in the measurement configuration, which generates a large, measured resistance value. While Figure 5c illustrates the actuator’s state without any current applied, Figure 5d showcases its movement subsequent to the application of 70 mA. A dynamic video of the EMA movement is provided in Video S1. Evidently, the actuator moves upward and exhibits substantial displacement even at low input currents. To accurately measure the displacement of the actuator, we utilized a white light interferometer (GCM-104M, Bruker, Karlsruhe, Germany). In this study, the actuator was subjected to various current levels, ranging from 10 mA to 100 mA, by connecting an external DC source. The experimental results obtained from this setup are presented in Figure 5e. The measurement error is due to the fact that the reflection of the sample surface affects the accuracy of the measurement when the white light interferometer is used. Although the actual displacement ultimately measured is small, the 250 μm is sufficient to combine metamaterials. 

The relationship between the electromagnetic force generated by the actuator and the current was measured using the mechanical probe (FT-RS1000, Femto Tools, Buchs, Switzerland). The actuator was connected to an external DC power supply, and the current was incrementally increased from 0 mA to 100 mA. The actual electromagnetic force was measured using a probe station on the device, and the results are presented in Figure 5f. The simulation curve is depicted by the black line, and the red line is the actual measurement. It should be noted that the manual movement of the probe station to the actuator center platform was necessary for measuring the electromagnetic force based on the deformation, which introduces a potential source of error. The dynamic time response of the actuator is characterized by a laser Doppler vibrometer (LDV, OFV-5000, Polytech Inc., Baden Württemberg, Germany), and the results are shown in Figure 5g,h. Figure 5g is the transient response when the actuator is turned on, and the time constant is approximately 0.084 ms. Figure 5h is the transient response when the actuator is turned off, and the time constant is 0.046 ms. The EMA exhibits approximately 4.3% variation for a constant driving current. The repeatability may be improved by hardening the stiffness of the movable plate.

**Figure 6:**
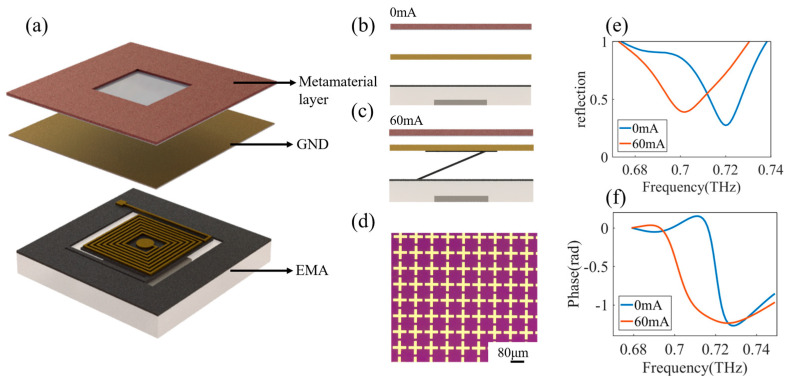
The EMA enabled tunable metamaterial absorber (TMA). (**a**) EMA combined metasurface diagram; (**b**) cross-section view of the TMA without current; (**c**) cross-section view of the TMA with 60 mA; (**d**) micrograph of metasurface structure; (**e**) reconfigurable TMA reflection spectrum; (**f**) reconfigurable TMA phase diagram.

To demonstrate the function of the flexible EMA, we developed a reconfigurable terahertz metamaterial absorber (TMA). Metamaterials are composed of subwavelength atoms that have great capabilities and flexibilities in controlling electromagnetic (EM) waves since their subwavelength meta-atoms can be designed and tailored in desired ways [31]. The TMA structure is usually composed of three layers: metamaterial layer, dielectric spacer, and ground plane layer [32]. TMA has great advantages in energy harvesting [33], refractive index sensing [34], imaging devices [35], and other applications. As shown in Figure 6a, we attached a metal ground plane (GND) on top of the actuator. The GND adopts copper-coated PI film with a PI thickness of 25 μm and a copper thickness of 2 μm. There is an air space between the GND and the metamaterial layer. The cross-section view of the TMA is shown in Figure 6b. By applying a current to the EMA, the GND is moving upward, and the separation distance between the GND and metamaterial layer decreases, as shown in Figure 6c. The metasurface structure is shown in Figure 6d; the pattern consists of a cross-shaped array with a length of 80 μm and a width of 10 μm. The instruments used to measure reflection and phase are time-domain terahertz spectral platforms (TERAFLASH PRO, TOPICA, Munich, Germany). It is in the reflection configuration with a beam diameter of 1 mm. The measured reflection amplitude and phase are shown in Figure 6e,f. At the frequency of 700 GHz, the modulation of the reflection coefficient is 0.5, and the phase modulation is approximately π/2.

We compared the performance of our EMA with previous studies, as listed in Table 2. We achieved 250 μm displacement by applying a current of 100 mA. The large displacement originated from the low stiffness of the flexible supporting beams, enabling large tunability of THz metamaterials.

## 5. Conclusions

In summary, we investigated the design and implementation of the EMA based on the flexible PI substrate. We optimized the structure of the EMA, including the planar spiral coil, the supporting beams, and the static magnets, by using the Multiphysics simulation. The designed EMA is fabricated by the combination of electroplating, etching, laser ablation, and assembling processes. The EMA may generate a force of 1.2 mN and displacement of 250 μm with an applied current of 100 mA. To demonstrate the function of the flexible EMA, we integrated it with a metamaterial structure to form a tunable terahertz metamaterial absorber. The EMA enabled an amplitude modulation depth of 0.5 and phase modulation of π/2 at 0.7 THz. The demonstration of flexible EMA holds great promise for the development of advanced electromagnetic wave control and manipulation technologies, opening new possibilities in various applications. The shortcoming of this paper is that the reliability of EMA is not analyzed. In future studies, we will focus on the reliability of EMA.

## Figures and Tables

**Table 1:** The geometric parameters of the proposed EMA.

Parameter	Value
Length of the side of the glass substrate (*L*)	8 mm
Thickness of glass substrate (*H*)	2 mm
Length of the side of the permanent magnet (*l*)	3 mm
Thickness of permanent magnet (*h*)	300 μm
Length of supporting cantilever beam (*L_BEAM_*)	4300 μm
Width of the supporting cantilever beam (*W_BEAM_*)	200 μm
Thickness of the supporting cantilever beam (*P*)	60 μm
Length of the side of the movable plate (*L_MP_*)	4 mm
Coil width (*W_wire_*)	10 μm
Coil thickness (*t_wire_*)	30 μm
Number of turns of the coil (*N*)	26

**Table 2:** The performance comparison between the present work and previous studies.

References	Materials	Drive Voltage or Current	Displacement
[36]	Silicon	8 mA	55 μm
[37]	Silicon	3–5 V	160 μm
[38]	Fe_3_O_4_	100 mA	65 μm
[39]	PDMS	30 mA	51.4 μm
[40]	Fe_3_O_4_	9 V	2.62 mm
[41]	Permalloy	250 mA	20 μm
This work	Polyimide	100 mA	250 μm

## Data Availability

All data and findings of this study are available from the corresponding authors upon reasonable request. The data are not publicly available due to privacy.

## Supplementary Materials

The following supporting information can be downloaded at: https://www.mdpi.com/article/10.3390/mi15020219/s1, Video S1: A dynamic video of the EMA movement.
